# Compensatory relationship between splice sites and exonic splicing signals depending on the length of vertebrate introns

**DOI:** 10.1186/1471-2164-7-311

**Published:** 2006-12-08

**Authors:** Colin N Dewey, Igor B Rogozin, Eugene V Koonin

**Affiliations:** 1 National Center for Biotechnology Information NLM, National Institutes of Health, Bethesda MD 20894, USA; 2 Department of Biostatistics and Medical Informatics, University of Wisconsin-Madison, USA

## Abstract

**Background:**

The signals that determine the specificity and efficiency of splicing are multiple and complex, and are not fully understood. Among other factors, the relative contributions of different mechanisms appear to depend on intron size inasmuch as long introns might hinder the activity of the spliceosome through interference with the proper positioning of the intron-exon junctions. Indeed, it has been shown that the information content of splice sites positively correlates with intron length in the nematode, Drosophila, and fungi. We explored the connections between the length of vertebrate introns, the strength of splice sites, exonic splicing signals, and evolution of flanking exons.

**Results:**

A compensatory relationship is shown to exist between different types of signals, namely, the splice sites and the exonic splicing enhancers (ESEs). In the range of relatively short introns (approximately, < 1.5 kilobases in length), the enhancement of the splicing signals for longer introns was manifest in the increased concentration of ESEs. In contrast, for longer introns, this effect was not detectable, and instead, an increase in the strength of the donor and acceptor splice sites was observed. Conceivably, accumulation of A-rich ESE motifs beyond a certain limit is incompatible with functional constraints operating at the level of protein sequence evolution, which leads to compensation in the form of evolution of the splice sites themselves toward greater strength. In addition, however, a correlation between sequence conservation in the exon ends and intron length, particularly, in synonymous positions, was observed throughout the entire length range of introns. Thus, splicing signals other than the currently defined ESEs, i.e., potential new classes of ESEs, might exist in exon sequences, particularly, those that flank long introns.

**Conclusion:**

Several weak but statistically significant correlations were observed between vertebrate intron length, splice site strength, and potential exonic splicing signals. Taken together, these findings attest to a compensatory relationship between splice sites and exonic splicing signals, depending on intron length.

## Background

Most protein-coding genes in multicellular eukaryotes are interrupted by multiple introns that are removed by the spliceosome in a complex succession of concerted hydrolysis and ligation reactions [1-4]. Obviously, high-precision recognition of introns is required for efficient splicing, and this recognition depends on an hierarchy of signals of varying specificity that are located both in the intron and in the exon and interact with different parts of the spliceosomal complex. The principal splicing signals are the donor and acceptor splice sites, the polypyrimidine tract preceding the acceptor site, and the branch point [5]. A consensus sequence ((A/C)AG|GU(A/G)AGU in vertebrates, the first two nucleotides of the intron are underlined) at the donor splice site is complementary to the 5' end of U1 small nuclear (sn)RNA, and the interaction between the donor site and U1 is thought to be the major requirement for splicing [6-8]. The CAG|G consensus sequence (the last two nucleotides of the intron are underlined) preceded by a polypyrimidine tract is typical of the acceptor splice site which is recognized by the splicing factor U2AF [9]. The branch point or, more precisely, the branch site is located upstream of the polypyrimidine tract preceding the acceptor site and consists of an A residue embedded in a distinct motif; this site is recognized by the U2 RNP and is involved in the formation of the lariat splicing intermediate [9,10].

However, the classical splice signals are insufficient for correct splicing, at least in vertebrates where intron lengths vary within an extremely broad range, and many introns are long. Moreover, it has been estimated that, while the canonical, intronic signals are sufficient for accurate splicing by "intron definition" in nematodes and flies, these signals contain only approximately one-half of the information required for correct splicing of even short introns in mammals and plants [11]. Additional splice signals have been identified in exons and can function by either facilitating (enhancers) or suppressing (silencers) splicing. Exonic splicing enhancers (ESE), in particular, appear to be present in most, if not all, mammalian exons, and have been characterized in considerable detail [12-15]. It is generally thought that the ESEs, which are located in the exon regions close to the donor or acceptor splice sites, promote the recognition of exons (often called exon definition) through binding SR proteins via their RRM domains, and thus facilitating the recruitment of the spliceosome to the vicinity of exon-intron junctions [16,17]. An inverse, compensatory relationship has been shown to exist between the strength of the splice sites in an exon and the dependence of splicing on ESEs, i.e., exons with weak, non-canonical splice sites, are strongly dependent on ESEs for their splicing [16]. Exonic splicing enhancers have been identified with a variety of experimental and computations methods, and combinations thereof [12,14,18-25]. In particular, the RESCUE-ESE method, the results of which have been extensively validated by mutational analysis of the predicted ESE motifs, has indicated that slightly more that 10% of the hexanucleotides in human exons could function as ESEs, reinforcing the notion that ESEs are very common in mammals [21,22]. The functional importance of ESEs has been further corroborated by the demonstration that the ESE hexanucleotides tend to be subject to purifying selection, i.e., single-nucleotide polymorphisms (SNPs) that disrupt ESEs in the vicinity of splice sites seem to be selected against [26].

A variety of observations indicate that the splicing process is not indifferent to intron length. Long introns might hinder the activity of the spliceosome through interference with the proper positioning of the intron-exon junctions. Thus, it could be expected that additional mechanisms and/or signals might be required for splicing of long introns. Indeed, it has been shown that the information content of splice sites positively correlates with intron length in the nematode [27], *Drosophila *[28], and fungi [29]. We were interested in developing an understanding of the effect of intron length on the evolution of the neighboring exons and the interaction of different types of splicing signals. Here, we describe the results of comparative analysis of vertebrate genes that reveal a complex interplay between splice sites and exonic signals depending on intron length.

## Results

### Long introns have stronger flanking splice sites than short introns

We first tested the hypothesis that long introns are associated with strong flanking splice sites in mammalian genomes. For each genome, we derived a position-specific weight matrix for both donor and acceptor splice sites. Using these matrices and background base frequencies, a log-odds score for each splice site was computed. The total splice site score for each intron was calculated as the sum of the scores of its flanking donor and acceptor sites.

**Figure 1:**
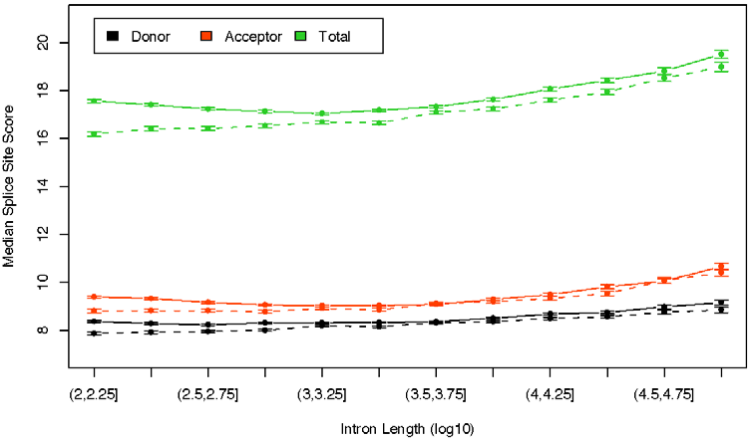
Donor, acceptor, and total splice site strength (in bits) increases with increasing intron length in human. Median standard error bars are given for different splice site scores over each range of intron lengths. Values for constitutive and alternative introns are shown with solid and dashed lines, respectively.

We found that, for human introns greater than 1.5 kb in length [the median length of human introns; (Figures S1, S2; see Additional File 1)], total splice site strength is positively correlated with intron length for both constitutive and alternative introns; the correlation coefficients were relatively small (constitutive *R *= 0.124, alternative *R *= 0.145) but highly statistically significant (*P *≈ 0) (Figure 1). Both the donor sites and, somewhat more prominently, the acceptor sites become stronger as the intron length increases. For introns shorter than 1.5 kb, constitutive and alternative introns displayed opposite, slight (constitutive *R *= -0.053, alternative *R *= 0.026) but statistically significant (constitutive P = 3.34e-34, alternative P = 6.27e-5) correlations between intron length and splice site strength (Figure 1). For almost all lengths, constitutive introns had significantly stronger splice sites than alternative introns (Mann-Whitney test). Mouse introns exhibited the same correlations as human introns (Figure S3; See Additional File 1).

### Long introns are associated with a higher A content in flanking exons leading to a greater density of potential ESEs

A second set of signals believed to be involved in the correct splicing of vertebrate introns are the ESEs, which tend to be located in the vicinity of the splice sites [21,23,30,31]. To determine if the strength of these signals is linked to intron length, we calculated the density of nucleotides within putative ESE sequences in the ends of exons flanking introns.

We searched exon ends for the hexamer ESE sequences identified by Fairbrother et al. [21]. The base composition of these putative ESEs is skewed relative to overall exon base frequencies, with the hexamers being composed of 48% A, 14% C, 25% G, and 13% T. Therefore, we also examined the base composition and expected number (through random shuffling of exon sequences) of ESE sites in each flanking exon.

**Figure 2:**
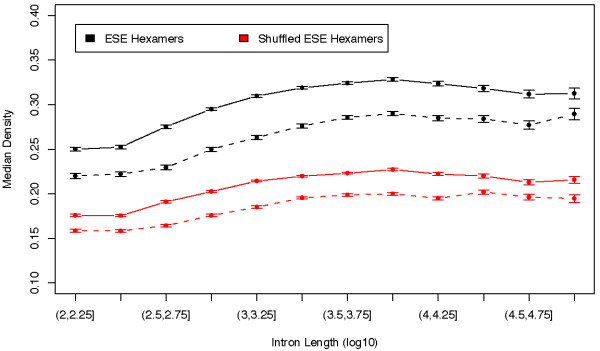
Densities of nucleotides occurring in sequences predicted to have ESE activity are correlated with intron length in human. Median standard error bars are plotted for each point. Values for constitutive and alternative introns are shown with solid and dashed lines, respectively.

**Figure 3:**
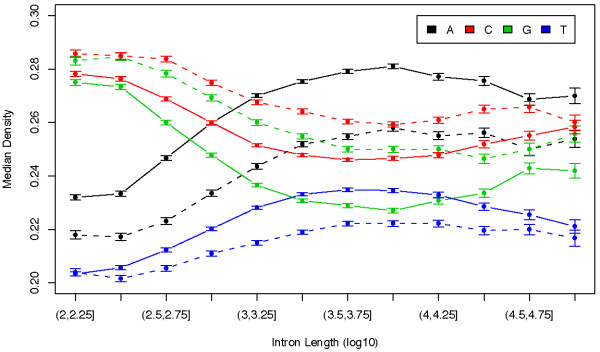
Nucleotide composition of exon ends flanking introns in human varies with intron length. Median standard error bars are plotted for each point. Values for constitutive and alternative introns are shown with solid and dashed lines, respectively.

Whereas for splice sites we observed correlations between signal strength and intron length for long introns (Figure 1), we found little, if any, correlation with ESE density for the same intron length range (Figure 2, Figure S4; see Additional File 1). However, for relatively short introns (<1.5 kb), we observed a positive correlation between intron length and the hexamer ESE density for both constitutive (*R *= 0.159, P ≈ 0) and alternative (*R *= 0.115, P ≈ 0) introns (Figure 2, Figure S4; see Additional File 1). As with splice site strength, ESE density was significantly higher in exons flanking constitutive introns than in those flanking alternative introns over all intron lengths (Mann-Whitney test). Since the putative ESE sequences have distinctive base compositions and variation in exon end base composition with intron length was observed (Figure 3, Figure S5; see Additional File 1), it seemed possible that the trends in ESE density were due entirely to the biases in base composition in exon ends. To test for this possibility, for each exon end, we calculated the difference (E_enrich_) between the observed ESE density (E_obs_) and the expected ESE density (E_expect_), given the base composition of that exon end. Note that E_enrich _is in terms of density, and is therefore normalized by exon end length. The resulting E_enrich _values had weaker but still significant correlations with intron length, for short constitutive (R = 0.086, P ≈ 0) and alternative (R = 0.060, P ≈ 0) introns. A slightly different test, in which the relative enrichment (E_enrich_/E_expect_) was calculated, yielded weaker correlation values for short constitutive (*R *= 0.012, P = 4.37e-3) and alternative (*R *= 0.011, P = 0.102) introns. The results of these tests suggest a specific increase in hexamer ESE density with increasing intron length among relatively short introns, as opposed to a more general trend in base composition.

### Differences in the lengths of orthologous introns are associated with corresponding changes in splicing signals

Having detected correlations between intron length and splicing signals for introns in a single genome, we sought to determine if differences in the lengths of orthologous introns in different species were associated with changes in splicing signals. As small changes in intron length are likely to have little effect on splicing, we examined only those pairs of orthologous introns for which the length difference was substantial (intron pairs in the upper and lower quartiles, with respect to percent length difference). When one intron of an orthologous pair was substantially longer than the other, and the various splice signals differed in strength, we compared the strengths signals associated with these introns.

Table 1 shows one such analysis, for human/chicken orthologous introns and total splice site scores. Table 2 shows *p*-values for chi-square tests performed on a variety of contingency tables such as that given in Table 1. The results clearly indicate that, on average, as the intron length in a particular site of orthologous genes increases during evolution, the splice signal strength increases in parallel.

### Higher sequence conservation and increased ESE density at exon ends

Although the exact locations of ESEs within exons are largely unknown, increased density of ESE motifs has been detected in the 25–30 nucleotides from exon ends, the regions of exons that have the lowest SNP density in humans [26]. We reproduced this test and confirmed the results in that the ESE density significantly increased in the vicinity of splice sites in both human (Figure S6; see Additional File 1) and mouse (Figure S7; see Additional File 1) exons.

**Figure 4:**
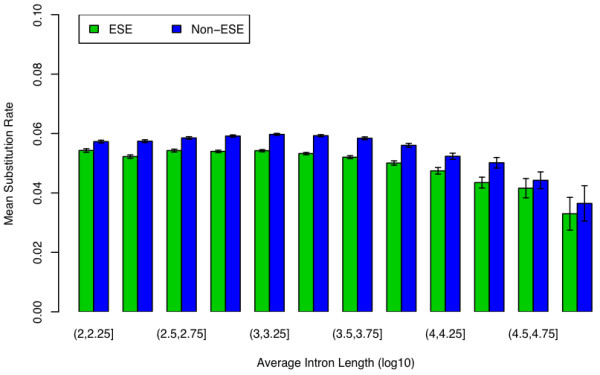
Mouse-rat substitution rates in exon ends for nucleotides inside and outside of ESE hexamers. Intron length was taken as the average of the mouse and rat intron lengths. Mean standard error bars are plotted for each substitution rate.

We further employed inter-species comparisons to examine the levels of sequence conservation at exon ends. In a comparison between mouse and rat, ESE sites were slightly but significantly more conserved than non-ESE sites (*P *≈ 0). ESE sites were substituted at a mean rate of 5.4% as compared to 5.9% for non-ESE sites. Excess conservation of ESE sites was found to occur over all intron lengths (Figure 4).

**Figure 5:**
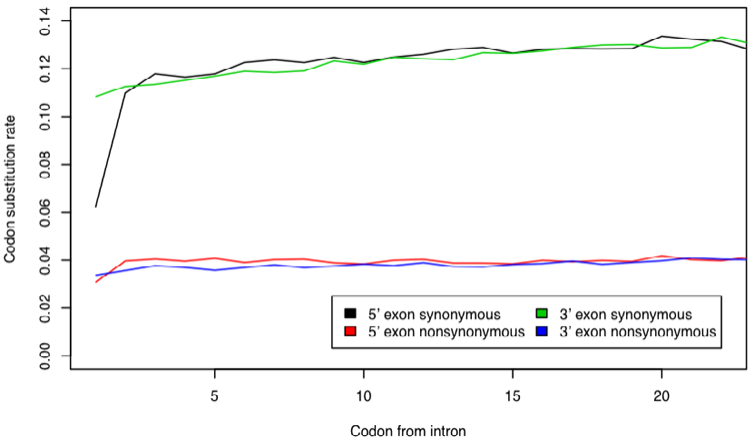
Synonymous and nonsynonymous codon substitution rates at the ends of exons flanking introns for mouse and rat. Synonymous substitutions were less frequent (*P *= 3e-85, chi-square test) within codons 3–12 than within codons 13–22, as counted from the nearest splice site (with codon 1 being the first whole codon inside of the exon). Non-synonymous substitutions exhibited a much weaker trend (*P *= 0.041).

Figure 5 shows the synonymous and nonsynonymous codon substitution rates at the ends of mouse and rat orthologous exons. Within 66 nucleotides (half of the median exon length) of splice sites, synonymous sites show a clear trend toward increased evolution rate with increased distance from the splice site whereas non-synonymous sites exhibited a very weak, if any, such trend. Conservation of exon ends between mouse and rat confirms similar results obtained with human SNPs [26]. Similar results supporting the notion that ESEs are functionally significant and evolve under purifying selection have been very recently reported by others [32].

### Longer introns are associated with higher conservation of synonymous and non-synonymous sites in flanking exons

**Figure 6:**
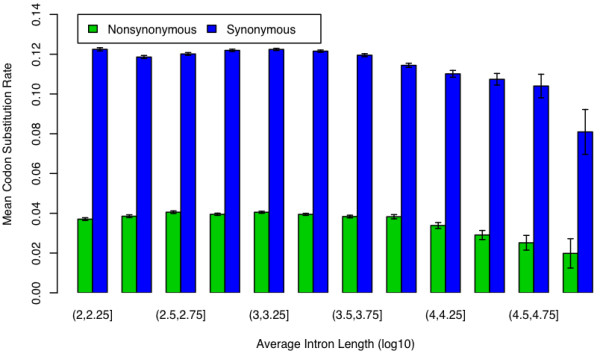
Rates of synonymous and nonsynonymous codon substitutions in exon ends over different lengths of the flanked intron. Codons split between exons were excluded from the analysis. The frequencies of both synonymous and nonsynonymous codon substitutions in exon ends flanking long introns (>1 kb, the median average mouse/rat intron length) show weak negative correlation (synonymous: *R *= -0.0546, *P *= 3.06E-27, nonsynonymous: *R *= -0.0297, *P *= 4.24E-9) with intron length. The data is for mouse/rat orthologous intron pairs. Mean standard error bars are given for each substitution rate.

If intron length is correlated with splicing signal strength, one would expect that sequences flanking long introns (sequences that might contain additional splicing signals) would be more conserved than those flanking short introns. Indeed, we found that both synonymous and nonsynonymous sites were more conserved in exon ends adjacent to long introns (Figure 6). The effect was notably more significant for the synonymous sites, which is compatible with the hypothesis that stronger sequence conservation in regions adjacent to long exons might be due to the presence of exonic splicing signals.

## Discussion and conclusion

The results presented here corroborate and extend previous observations on the connection between intron length and the strength of splicing signals. Generally, we observed that, as expected, longer introns that might present greater problems for intron definition and, consequently, for efficient splicing than shorter introns were associated with stronger splicing signals. However, there was a notable compensatory relationship between different types of signals, namely, the splice sites and the ESEs. In the range of relatively short introns (approximately, < 1.5 kb in length), the enhancement of the splicing signals in longer introns seemed to occur within the exons and was manifest in the increased concentration of ESEs. In contrast, for longer introns, this effect was not detectable, and what was seen instead, was an increase in the strength of the donor and acceptor splice sites. Since the ESEs are located in protein-coding exons, it appears likely that accumulation of A-rich hexamers beyond a certain limit is incompatible with functional constraints operating at the level of protein sequence evolution. Hence the compensation in the form of evolution of the splice sites themselves toward greater strength.

The threshold separating "short" and "long" introns used here was different from the boundary at ~200 nucleotides that separates introns spliced via intron definition from those spliced via the exon definition [33,34]. We also repeated the analyses described in the text with an additional partition of short introns at the 200 nucleotide cut-off. The "intron-defined" short introns (<200 nucleotides) were found to have correlations of the same sign as those that are "exon defined" (200 to 1500 nucleotides) but of lower significance (data not shown). Thus, more complex phenomena seem to be at play than, simply, the distinction between the intron and exon definitions. Apparently, even among introns that are spliced via the exon definition, the relative contributions of the splice sites and additional, exonic splicing signals depend on the length of the intron.

All of the above relationships consistently held for both constitutive and alternative exons. Curiously, however, the connection between splice site strength and intron length was somewhat stronger for the alternative exons whereas the opposite was true of the dependence between ESE density and intron length, which was most pronounced for constitutive exons. This suggests yet another level of compensation between different types of splicing signals.

The correlations between intron length and ESE density that were readily observable for ESE hexamers, were not seen when we analyzed a different class of ESEs that have been defined as octamer motifs [23,24] (Figures S8 and S9; see Additional File 1). However, this observation is somewhat hard to interpret because the octamer ESEs have been identified in non-coding exons [23]. Thus, it remains to be determined whether the octamer ESEs are less important in coding than in non-coding exons or they function in a manner different from that of the hexamer exons and independent of intron length.

In addition to the above dependences, we observed a correlation between intron length and sequence conservation in the exon ends, especially, in synonymous positions, throughout the entire length range of introns (Figure 6). This suggests that splicing signals other than the ESE hexamers, which were originally defined for short exons [21], or the ESE octamers [23,24] analyzed here, might exist in exon sequences, particularly, those that flank long introns; such signals that remain to be specifically characterized, might comprise a new class of ESEs.

The greater strength of different types of splicing signals around longer introns could evolve either via a neutral evolution route or as an adaptation. The neutral scenario would apply to a case when an intron becomes shorter as a result of deletion that is followed by splice site amelioration. In contrast, when the length of an intron increases due to an insertion, the splicing signals would evolve under positive selection, adapting to the new situation by restoring splicing efficiency.

All the trends in the relationships between the strength of splicing signals, evolutionary conservation of synonymous positions in exon ends and ESE sites, and intron length that are reported here and elsewhere [6-8] are manifest in weak correlations that are made statistically (highly) significant by the large, genome-wide size of the analyzed samples of exons and introns. On the one hand, this illustrates the power of genome-scale analysis in detecting subtle but potentially functionally relevant signals in sequences. On the other hand, the dependence of these observations on the vast amounts of data makes the analysis susceptible to systematic biases in that data, e.g., those in nucleotide composition. We attempted to eliminate the potential effects of such biases whenever we could discern them and found the connections withstood the controls, even though some correlations were weakened. The weak correlations between intron size and splice signal strength and exonic sequence features (including ESE density and conservation) suggest that intron definition is governed by multiple signals, some of which remain to be recognized. In particular, it seems likely that some of the signals that are important for efficient splicing of exons flanking long introns reside within those introns. This possibility seems to be compatible with the recent findings that long introns show a greater evolutionary conservation than short introns in Drosophila [35] and that mammalian long introns are enriched in multispecies conserved sequence elements compared to short introns [36].

On balance, despite the weakness of the observed correlations, the coherence of different types of signals uncovered in this study, some of which are limited to single-genome analysis (splice site strength and ESE density) whereas others involve sequence conservation in different genomes, strongly suggests that these observations are functionally relevant for the splicing mechanism rather than spurious. In particular, these findings provide an incentive for an experimental search for new types of ESEs.

## Methods

### Single species analysis

We used the NCBI build 34 assembly (July 2003) of the human genome and the NCBI build 33 assembly (May 2004) of the mouse genome. For single species analyses, intron annotations were obtained from the Alternative Splicing Database (ASD) [37]. Human Release 1 and Mouse Release 1 of this database were used. Confirmed introns from the database were categorized as "alternative" if they overlapped with another intron of the same locus or were involved in a "retention" event (i.e., the entire intron was part of an exon in an alternate transcript). Introns not marked as "alternative" were considered "consititutive." After filtering out introns with Ns and those with non-standard splice sites, a total of 153,744 introns from 14,819 human genes and 134,037 introns from 15,018 mouse genes were analyzed. The median intron length was 1,557 bp for human and 1,185 bp for mouse (Figures S1, S2; see Additional File 1).

Position-specific weight matrices were constructed for both donor and acceptor splice sites [38]. A donor splice site was taken to be the last 3 bases of the 5' exon and the first 6 bases of the intron. An acceptor splice site was taken to be the last 20 bases of the intron and the first base of the 3' exon. Log-odds scores (in bits) were computed for each splice site, defined as ∑log⁡2pi,s(i)qs(i)
 MathType@MTEF@5@5@+=feaafiart1ev1aaatCvAUfKttLearuWrP9MDH5MBPbIqV92AaeXatLxBI9gBaebbnrfifHhDYfgasaacH8akY=wiFfYdH8Gipec8Eeeu0xXdbba9frFj0=OqFfea0dXdd9vqai=hGuQ8kuc9pgc9s8qqaq=dirpe0xb9q8qiLsFr0=vr0=vr0dc8meaabaqaciaacaGaaeqabaqabeGadaaakeaadaaeabqaaiGbcYgaSjabc+gaVjabcEgaNnaaBaaaleaacqaIYaGmaeqaaaqabeqaniabggHiLdGcdaWcaaqaaiabdchaWnaaBaaaleaacqWGPbqAcqGGSaalcqWGZbWCcqGGOaakcqWGPbqAcqGGPaqkaeqaaaGcbaGaemyCae3aaSbaaSqaaiabdohaZjabcIcaOiabdMgaPjabcMcaPaqabaaaaaaa@4274@, where *p*_*i,j *_is the frequency of nucleotide *j *at splice site position *i*, *q*_*j *_is the background transcript frequency of nucleotide *j*, and *s*(*i*) denotes the nucleotide at position *i *within splice site *s*. The background frequencies of nucleotides within human and mouse RefSeq pre-mRNA transcripts were found to be 27.5% A, 20.8% C, 21.6% G, and 30.1% T.

Putative hexamer ESE sequences for human and mouse, as determined by Fairbrother et al. [21], were obtained from the RESCUE-ESE [22] Web site [39]. The number of putative ESE hexamers predicted by RESCUE-ESE and employed for the present analysis was 238 for human and 508 for mouse. An exon nucleotide was considered to be an ESE site if any substring containing that nucleotide was one of the putative ESE sequences. To calculate the expected number of ESE sites found in an exon end, given its base composition, the sequence of the exon end was randomly shuffled 100 times and the expected number of ESE sites was taken to be the mean over these 100 shuffled sequences. The end of an exon was defined as 100 bases from the intron/exon junction, or half of the exon if it was less than 200 bases in length. Thus, a base was never considered as part of both the 5' and 3' exon ends.

### Comparative analysis

For comparative analyses, the RefSeq [40] mRNA data set was used instead of the ASD data set in order to have coding information for exons flanking the analyzed introns. Mappings of RefSeq mRNAs to the human and mouse genome sequences were obtained from the UCSC Genome Browser [41]. RefSeq mapped gene structures were filtered for those sequences that began with a start codon, ended with a stop codon, and did not contain an internal stop codon. A total of 154,986 unique introns from 23,662 human RefSeq genes, and 113,865 unique introns from 16,346 mouse RefSeq genes were analyzed. Only introns that were flanked by two coding exons were considered in this analysis. We additionally removed duplicate introns that appear in the RefSeq data set due to the inclusion of alternatively spliced mRNAs. An intron was classified as "short" if its length was less than the median length, or "long" otherwise.

Orthologous intron pairs were obtained through the use of a 9 vertebrate (human, chimp, mouse, rat, dog, chicken, fugu, tetraodon, zebrafish) multiple whole-genome alignment, obtained from [42]. This alignment was constructed by a combination of Mercator [43], an orthology mapping program, and MAVID [44], a multiple alignment program for long genomic sequences. Given the coordinates of a RefSeq intron in human or mouse, the coordinates of the introns orthologous to it in the other species were obtained by identifying the intervals aligned to its flanking exons in the multiple whole-genome alignment. A mapping of a reference intron was considered to be valid if (1) its flanking exons mapped to the same chromosome and strand in the target genome, (2) its flanking exons mapped in the same order and orientation as in the reference genome, (3) the lengths of the mapped exons were equal to those of the reference exons, (4) the mapped intron started with "GT" and ended with "AG," (5) no internal stop codons were found in the mapped exons, (6) the length of the intron was at least 100 bp in both species, and (7) neither intron sequence contained the character N (the presence of this character often indicates uncertainty of length).

For the purpose of measuring changes in splice site scores of orthologous introns, position-specific weight matrices were constructed using splice sites from both genomes under consideration. When calculating contingency tables, such as that shown in Table 1, only orthologous intron pairs with large length differences and different splice site strengths were considered. An orthologous intron pair was said to have a large length difference if its percent length difference, length1−length2(length1+length2)/2
 MathType@MTEF@5@5@+=feaafiart1ev1aaatCvAUfKttLearuWrP9MDH5MBPbIqV92AaeXatLxBI9gBaebbnrfifHhDYfgasaacH8akY=wiFfYdH8Gipec8Eeeu0xXdbba9frFj0=OqFfea0dXdd9vqai=hGuQ8kuc9pgc9s8qqaq=dirpe0xb9q8qiLsFr0=vr0=vr0dc8meaabaqaciaacaGaaeqabaqabeGadaaakeaadaWcaaqaaiabdYgaSjabdwgaLjabd6gaUjabdEgaNjabdsha0jabdIgaOnaaBaaaleaacqaIXaqmaeqaaOGaeyOeI0IaemiBaWMaemyzauMaemOBa4Maem4zaCMaemiDaqNaemiAaG2aaSbaaSqaaiabikdaYaqabaaakeaacqGGOaakcqWGSbaBcqWGLbqzcqWGUbGBcqWGNbWzcqWG0baDcqWGObaAdaWgaaWcbaGaeGymaedabeaakiabgUcaRiabdYgaSjabdwgaLjabd6gaUjabdEgaNjabdsha0jabdIgaOnaaBaaaleaacqaIYaGmaeqaaOGaeiykaKIaei4la8IaeGOmaidaaaaa@5799@, was in the lower or upper quartile. Therefore, roughly one-half of the intron pairs in each contingency table have the length of the intron in the first genome less than the length of the intron in the second genome.

### Statistical analyses

Statistical analyses were performed using the R package [45]. All correlation statistics were calculated using Pearson's test. Chi-squared statistics were computed using Pearson's Chi-squared test with Yates' continuity correction.

## Authors' contributions

CND participated in the data collection, performed most of the data analysis and wrote the first draft of the manuscript; IBR participated in the data collection and study design, and performed some of the statistical analyses; EVK initiated the study, participated in its design and the interpretation of the results, and wrote the final version of the manuscript. All authors read and approved the final version of the manuscript.

## Supplementary Material

Additional File 1 The complete contingency table used in this analysis and Figures S1-9. Click here for file

## Figures and Tables

**Table 1:** A contingency table used to test whether there are associations between changes in intron length and changes in splice site scores, for human/chicken orthologous intron pairs.

		**Total Splice Site Score**	
		human < chicken	human > chicken
**Intron Length**	human < chicken	7024	5106
	human > chicken	5943	6210

The chi-square test gives a *p*-value of 7.55E-45 for this table, indicating that there is a significant association between changing intron length and changing splice site signals.

**Table 2:** Differences in intron length of orthologous intron pairs are correlated with differences in splice sites scores and ESE and A-nucleotide density

	**Human/Chimp**	**Human/Mouse**	**Human/Rat**	**Human/Dog**	**Human/Chicken**	**Mouse/Rat**	**Mouse/Dog**
**Total splice site score**	9.19E-6	5.31E-9	2.02E-6	2.33E-3	2.64E-43	0.104	6.31E-5
**Donor score**	1	8.79E-3	1.38E-4	0.0485	4.96E-25	1	2.33E-6
**Acceptor score**	1.75E-6	2.33E-8	3.17E-3	0.521	2.96E-25	1	8.52E-3
**ESE sites**	1	3.49E-3	0.0149	7.80E-4	2.10E-29	1	3.45E-4
**A-content**	1	1.62E-4	1.08E-06	5.89E-9	2.28E-83	1.45E-3	5.37E-28

In all significant cases, a larger intron tended to correspond with stronger signals. The table shows Bonferroni-corrected (*n *= 35) *p*-values for chi-square tests performed on 2 by 2 contingency tables of the form shown in **Table 1**.
