# Supplementary material for: Compensatory relationship between splice sites and exonic splicing signals depending on the length of vertebrate introns
Source: BMC Genomics. 2006 Dec 8;7:311. doi: 10.1186/1471-2164-7-311 (PMC1713244; doi:10.1186/1471-2164-7-311)
Supplement: Additional File 1 — The complete contingency table used in this analysis and Figures S1-9. [file 1471-2164-7-311-S1.doc]

**Supplemental Material for:**

**Compensatory relationship between splice sites and exonic splicing signals depending on the length of vertebrate introns**

Colin N. Dewey^¶^, Igor B. Rogozin, and Eugene V. Koonin*

National Center for Biotechnology Information NLM, National Institutes of Health, Bethesda MD 20894, USA

^¶^Present address: Department of Biostatistics and Medical Informatics, University of Wisconsin-Madison

*Correspondence to: Eugene V. Koonin, National Center for Biotechnology Information NLM, National Institutes of Health, Bethesda MD 20894, USA; Tel.: 301.435.5913; Fax: 301.435.7794; Email: [koonin@ncbi.nlm.nih.gov](mailto:koonin@ncbi.nlm.nih.gov)

Supplemental table S1

The contingency tables used to test for associations between changes in intron length and changes in splice site scores, ESE sites, and A-content. The table in the “Total SS score” row and “Human/Chicken” column is the same as that given in Table 1 and the layouts of the other tables are defined similarly. The significance of each table is reported in Table 2.

|  | Human/Chimp | | Human/Mouse | | Human/Rat | | Human/Dog | | Human/Chicken | | Mouse/Rat | | Mouse/Dog | |
| --- | --- | --- | --- | --- | --- | --- | --- | --- | --- | --- | --- | --- | --- | --- |
| Total SS score | 2487 | 2268 | 13140 | 11568 | 12046 | 10594 | 13277 | 12744 | 7024 | 5106 | 9207 | 8952 | 8652 | 8791 |
|  | 2205 | 2488 | 12444 | 12296 | 11481 | 11184 | 12844 | 13223 | 5943 | 6210 | 8926 | 9239 | 8203 | 9235 |
| Donor score | 408 | 400 | 8384 | 7278 | 7809 | 6701 | 6701 | 6302 | 5652 | 4812 | 3393 | 3227 | 6012 | 5936 |
|  | 327 | 358 | 8206 | 7738 | 7576 | 7244 | 6782 | 6900 | 4888 | 5594 | 3322 | 3373 | 5537 | 6292 |
| Acceptor score | 2174 | 1946 | 12891 | 11716 | 11697 | 10858 | 12998 | 12747 | 6993 | 5112 | 8822 | 8717 | 8683 | 8714 |
|  | 1928 | 2197 | 12228 | 12424 | 11294 | 11288 | 12758 | 13062 | 6184 | 5964 | 8617 | 8922 | 8336 | 9053 |
| ESE sites | 1977 | 2018 | 11462 | 11439 | 10457 | 10457 | 11527 | 11570 | 6989 | 4568 | 8297 | 8243 | 8174 | 8334 |
|  | 1947 | 2035 | 11051 | 11863 | 10123 | 10847 | 11175 | 12138 | 6167 | 5482 | 8260 | 8386 | 7806 | 8774 |
| A-content | 2523 | 2584 | 9598 | 11971 | 8830 | 10974 | 10386 | 11085 | 7110 | 4001 | 7478 | 7243 | 8553 | 6628 |
|  | 2506 | 2529 | 9092 | 12399 | 8281 | 11518 | 9847 | 11890 | 5598 | 5380 | 7094 | 7563 | 7602 | 7640 |


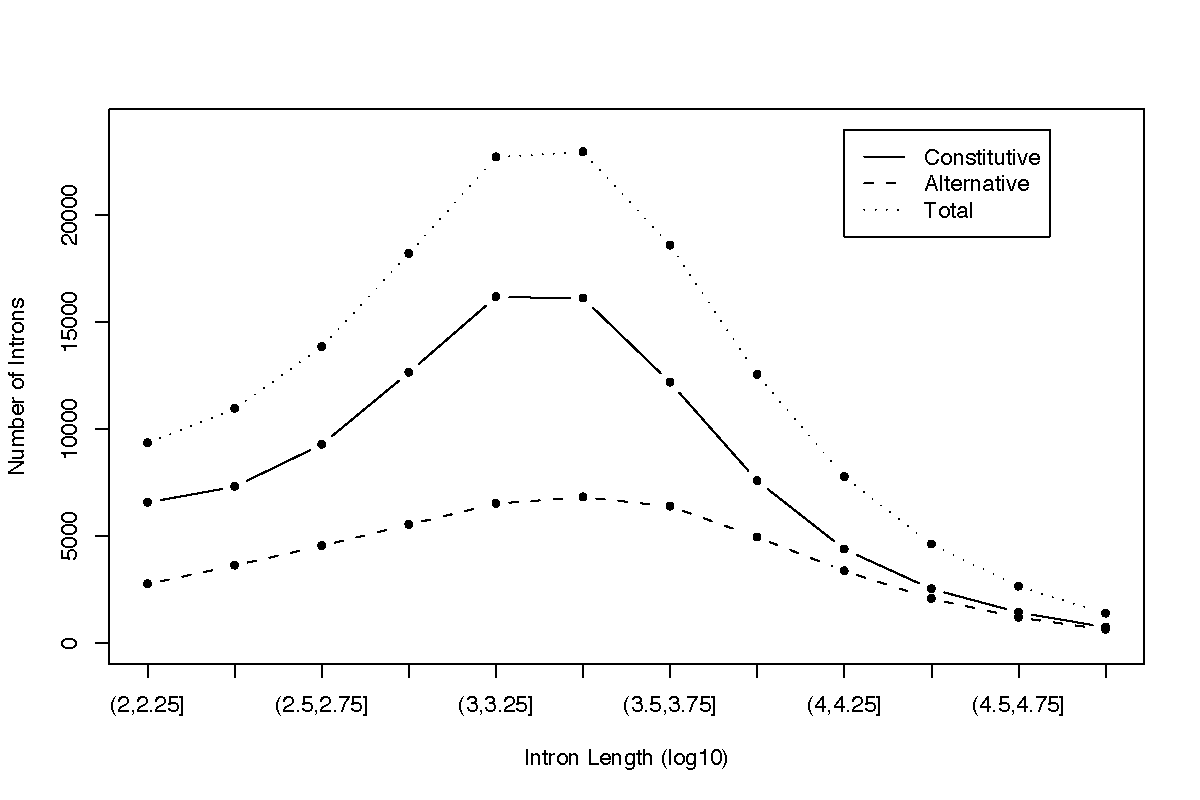


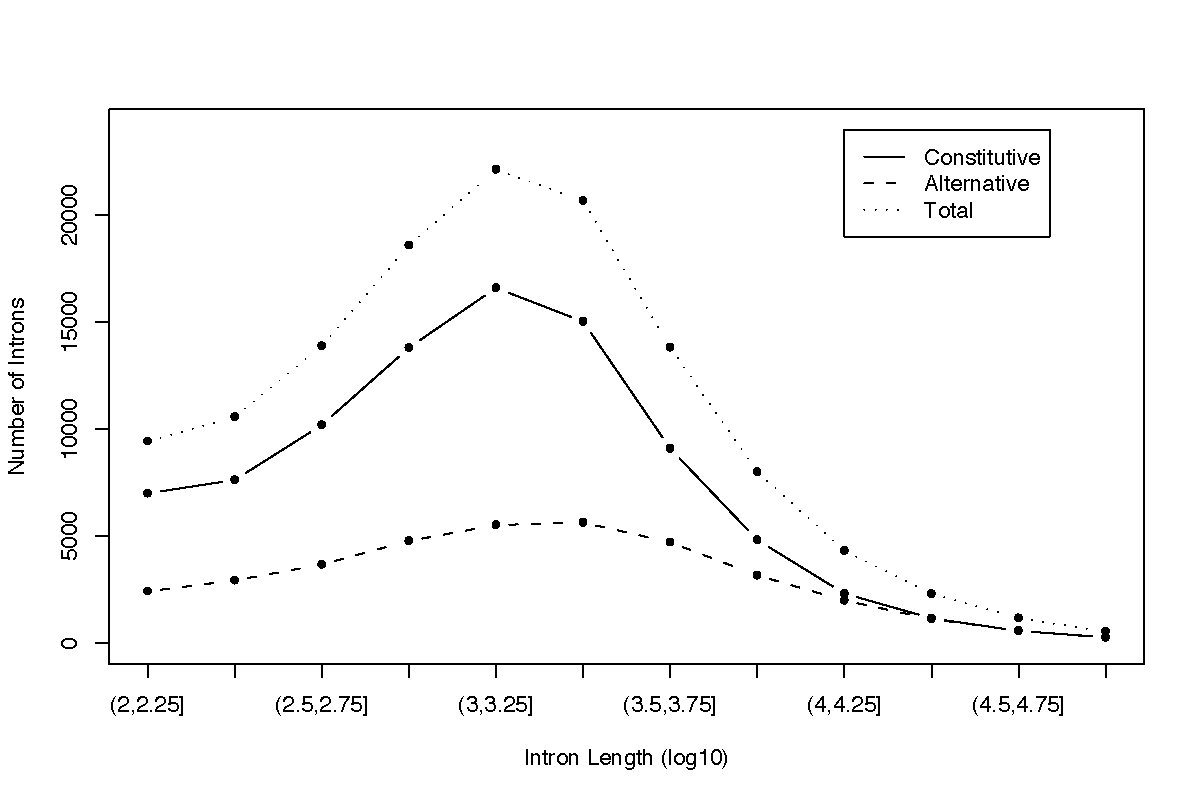


Figure S 2


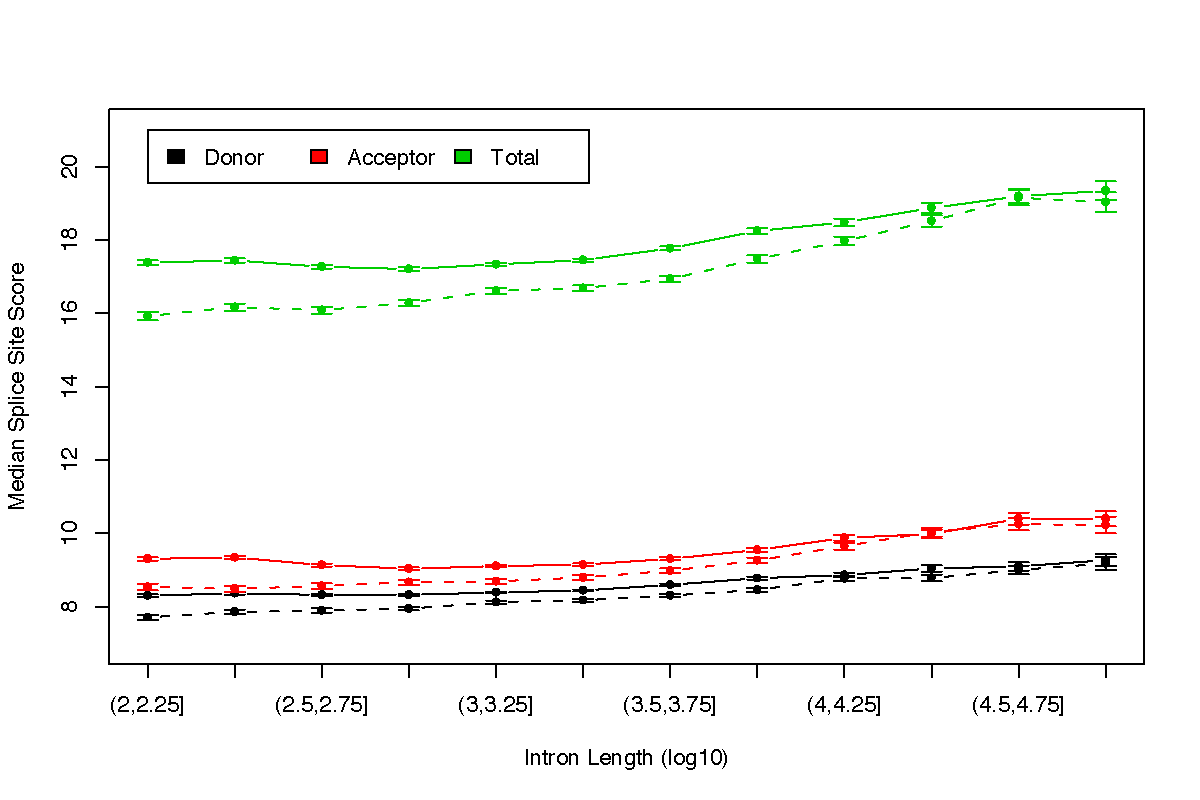


Figure S3


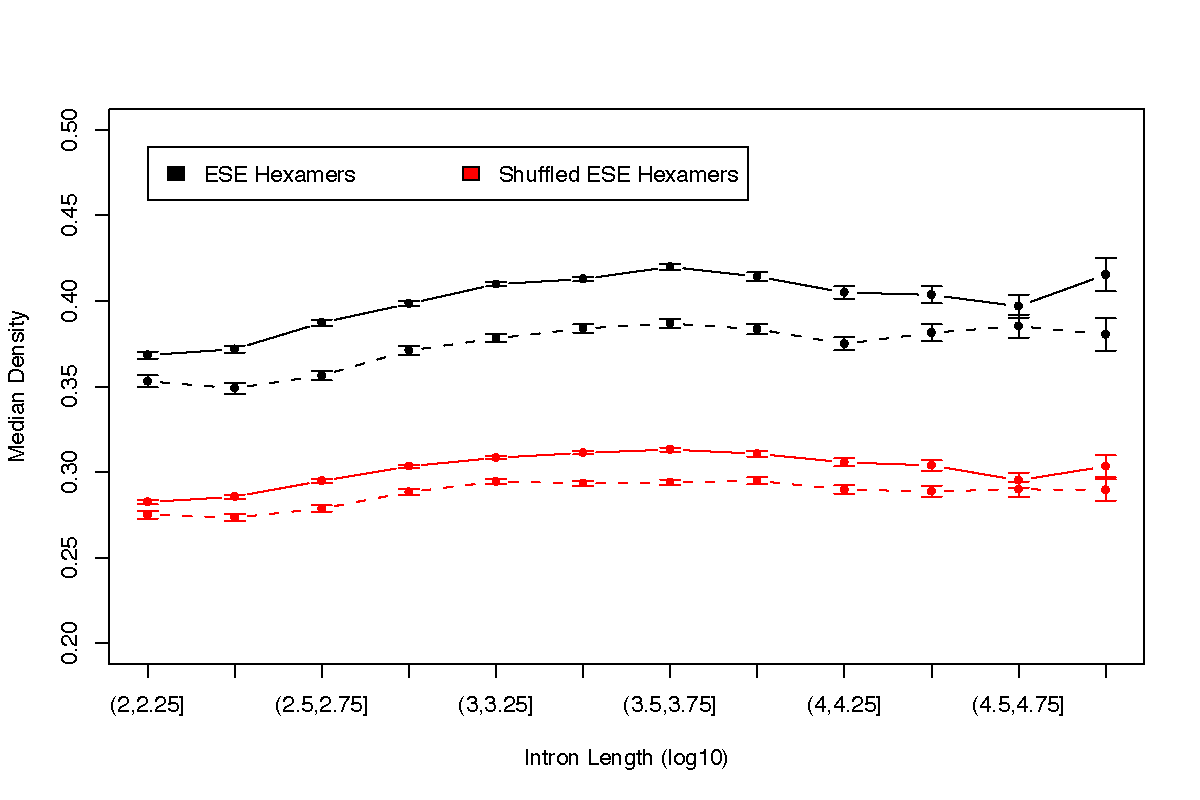


Figure S4. Nucleotide composition of exon ends flanking introns in mouse varies with intron length. Median standard error bars are plotted for each value. Values for constitutive and alternative introns are shown with solid and dashed lines, respectively.


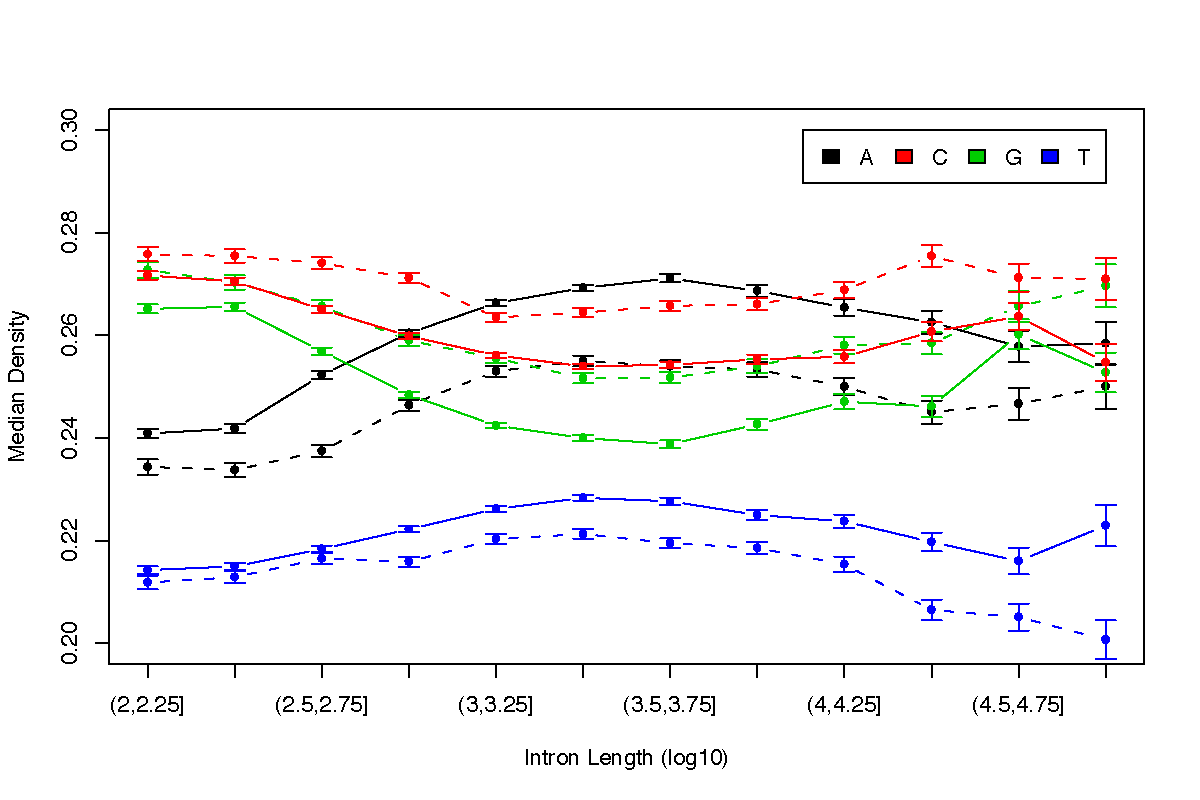


Figure S6


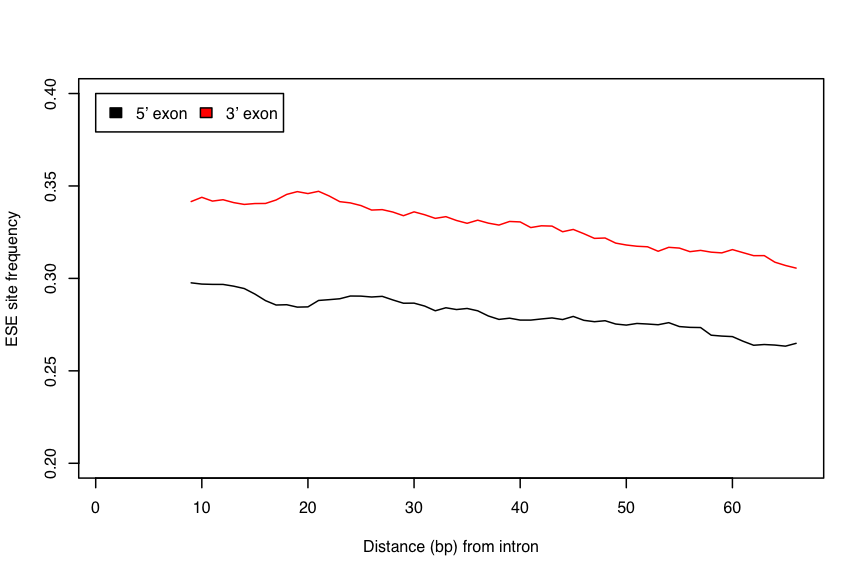


Figure S6


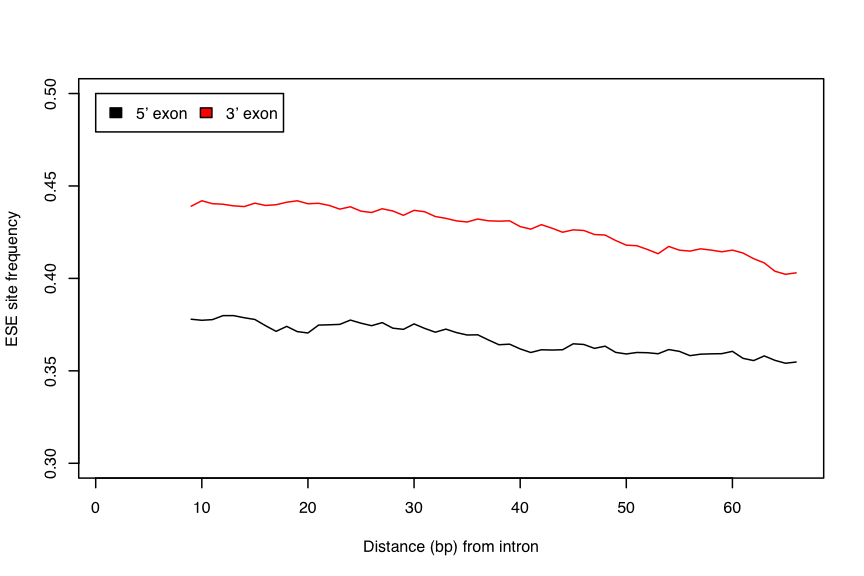


Figure S7
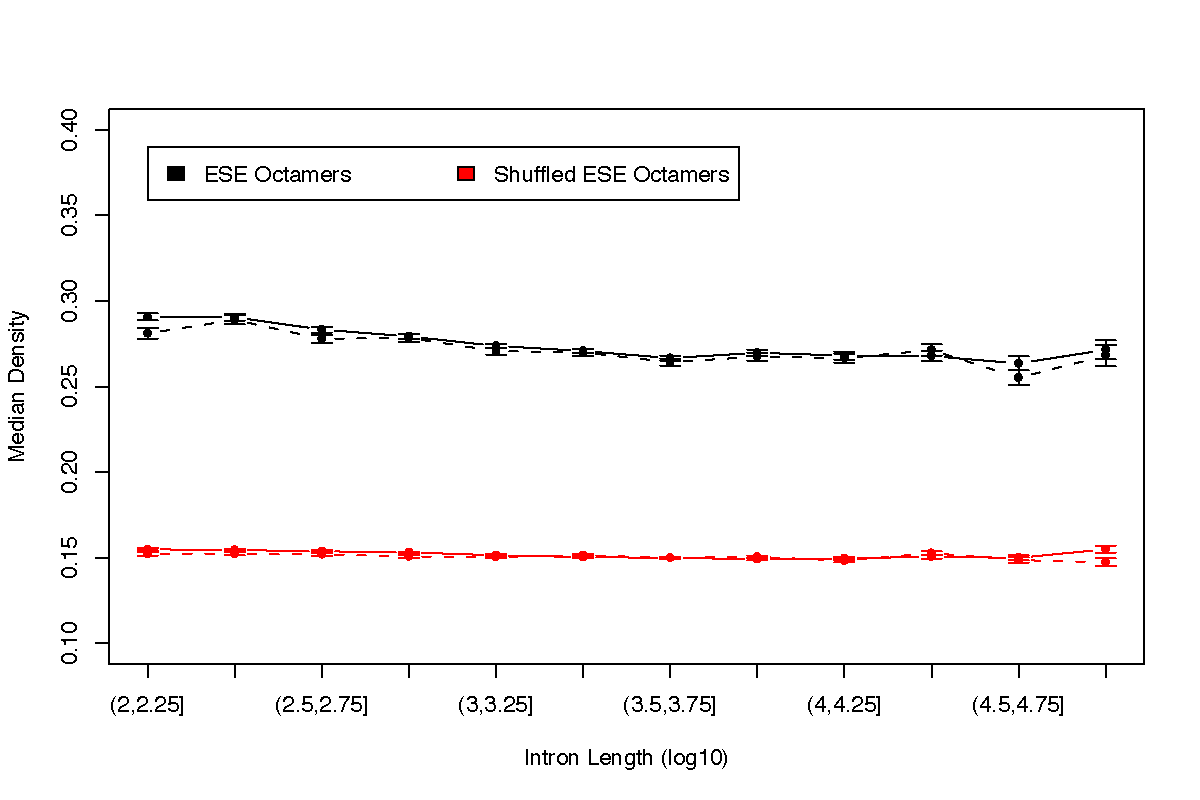


Figure S8


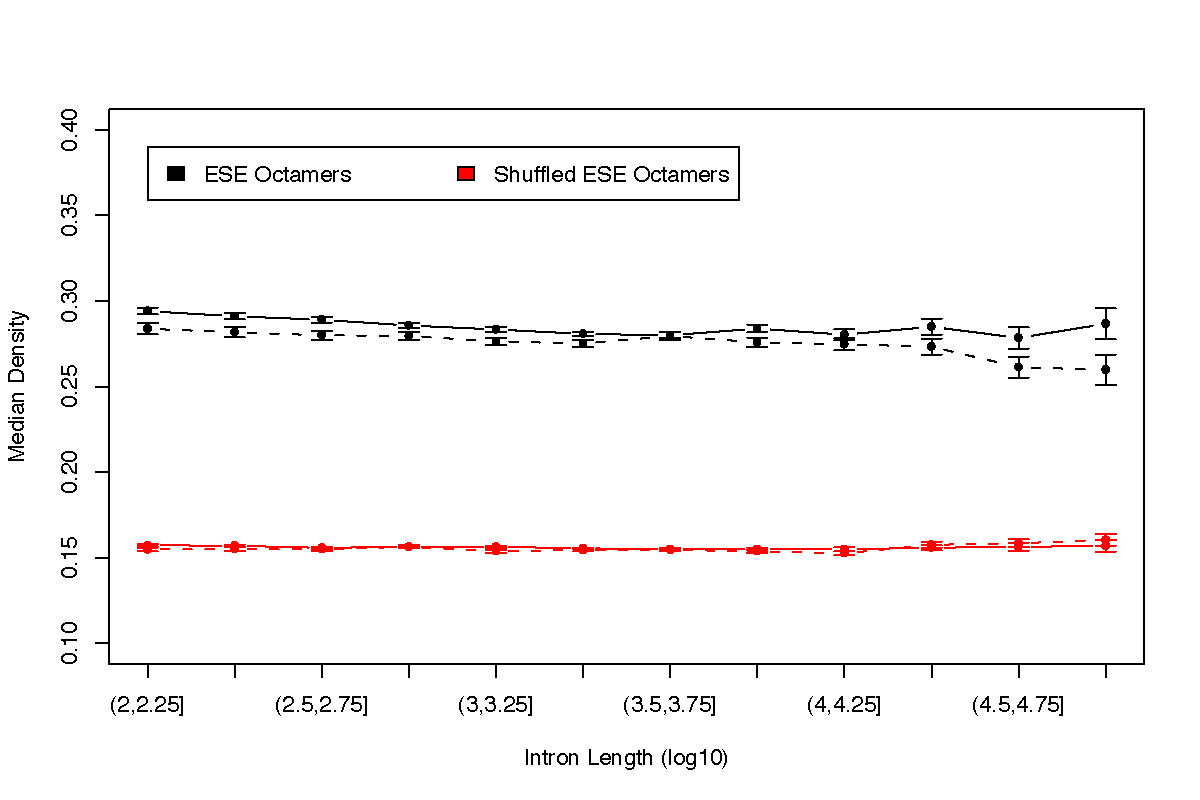


Figure S9

Supplemental figure legends

Figure S 1. Distribution of human intron lengths.

Figure S 2. Distribution of mouse intron lengths.

Figure S3. Splice site strength increases with increasing intron length in mouse.

As in human, a significant positive correlation (constitutive: *R* = 0.108, *P* ≈ 0, alternative: *R =* 0.161, P ≈ 0) between intron length and splice site strength occurs for long introns (≥1.2kb), whereas short introns (<1.2kb) have a very weak correlation with splice site strength (constitutive: *R* = -0.028, *P* = 3.26e-10, alternative: *R* = 0.015, P = 0.0493). Median standard error bars are plotted for each value. Values for constitutive and alternative introns are shown with solid and dashed lines, respectively.

Figure S4. Nucleotide composition of exon ends flanking introns in mouse varies with intron length. Median standard error bars are plotted for each value. Values for constitutive and alternative introns are shown with solid and dashed lines, respectively.

Figure S5. Densities of nucleotides occurring in sequences predicted to have ESE activity are correlated with intron length in mouse.

For introns of length less than 1.2kb, a significant positive correlation (constitutive: *R* = 0.112, *P* ≈ 0, alternative: *R* = 0.082, P ≈ 0) between intron length and hexamer ESE nucleotide density is observed. Median standard error bars are plotted for each value. Values for constitutive and alternative introns are shown with solid and dashed lines, respectively.

Figure S6. Frequency of human ESE hexamer sites as a function of distance from the nearest intron.

ESE sites are significantly (*P* ≈ 0, chi-square test) more frequent within bases 11-38 than within bases 39-66, as counted from the nearest splice site.

Figure S7. Frequency of ESE hexamer sites is highest at the ends of mouse exons.

ESE sites are significantly (*P* ≈ 0, chi-square test) more frequent within bases 11-38 than within bases 39-66, as counted from the nearest splice site.

Figure S8. Densities of human exon nucleotides occurring in ESE octamers

Values for constitutive and alternative introns are shown with solid and dashed lines, respectively.

Figure S9. Densities of mouse exon nucleotides occurring in ESE octamers .

Values for constitutive and alternative introns are shown with solid and dashed lines, respectively.
